# Correction: Rheu et al. Effect of Fermented Sarco Oyster (*Crassostrea gigas*) Extract on Muscle Strength Enhancement in Postmenopausal Females: A Randomized, Double-Blind, Placebo-Controlled Trial. *Int. J. Environ. Res. Public Health* 2022, *19*, 16450

**DOI:** 10.3390/ijerph20217011

**Published:** 2023-11-02

**Authors:** Kyoung-Min Rheu, Bae-Jin Lee, Woo-Hyeon Son, Dong-Seok Kim, Hyun-Tae Park, Min-Seong Ha, Byong-Hak Gong, Byeong-Hwan Jeon

**Affiliations:** 1 Marine Bioprocess Co., Ltd., Busan 46048, Republic of Korea; kmin.rheu@gmail.com (K.-M.R.); hansola82@hanmail.net (B.-J.L.); 2 Institute of Convergence Bio-Health, Dong-A University, Busan 49236, Republic of Korea; physical365@gmail.com; 3 Department of Sports and Health Science, Kyungsung University, Busan 48434, Republic of Korea; dongseok@me.com; 4 Graduate School of Health Care and Sciences, College of Health Science, Dong-A University, Busan 49236, Republic of Korea; htpark@dau.ac.kr; 5 Department of Sports Culture, Dongguk University, Seoul 04620, Republic of Korea; haminseong@uos.ac.kr; 6 Korea Sports Culture Association, Busan 04420, Republic of Korea; gongbk@hanmail.net

There was an error in the original publication [1]. The BMI values were incorrectly written as ranging between 5 and 30.0 kg/m^2^, when the correct range is between 18.5 and 30.0 kg/m^2^.

A correction has been made to the first paragraph of Section 2. Materials and Methods, 2.2. Participant Eligibility.

Participants aged 65 years or older, female, with a body mass index (BMI) between 18.5 and 30.0 kg/m^2^, and with a relatively low skeletal muscles mass (<110% of the standard lean mass) were eligible for the study. Participants with abnormal liver or renal function (aspartate aminotransferase (AST) or alanine aminotransferase (ALT) ≥ 60 IU/L, creatinine level ≥ 1.2 mg/dL, urinalysis dipstick reading of ≥2+), uncontrolled hypertension (blood pressure (BP) ≥ 160/100 mmHg), uncontrolled hyperthyroidism or hypothyroidism, uncontrolled diabetes (fasting glucose level ≥ 160 mg/dL), a history of gastrectomy, mental disorder, known allergies, addiction to alcohol or drugs, and those with a notable cardiovascular disease or central bone fracture within the past 6 months were excluded. Fifty-two females participated in our study.

The authors apologize for any inconvenience caused and state that the scientific conclusions are unaffected. The original publication has also been updated.
