# Supplementary material for: Effect of Fermented Sarco Oyster (Crassostrea gigas) Extract on Muscle Strength Enhancement in Postmenopausal Females: A Randomized, Double-Blind, Placebo-Controlled Trial
Source: Int J Environ Res Public Health. 2022 Dec 8;19(24):16450. doi: 10.3390/ijerph192416450 (PMC9779144; doi:10.3390/ijerph192416450)
Supplement: Supplementary file 1 [file ijerph-19-16450-s001.zip › ijerph-2053744-supplementary.pdf]

Supplementary materials

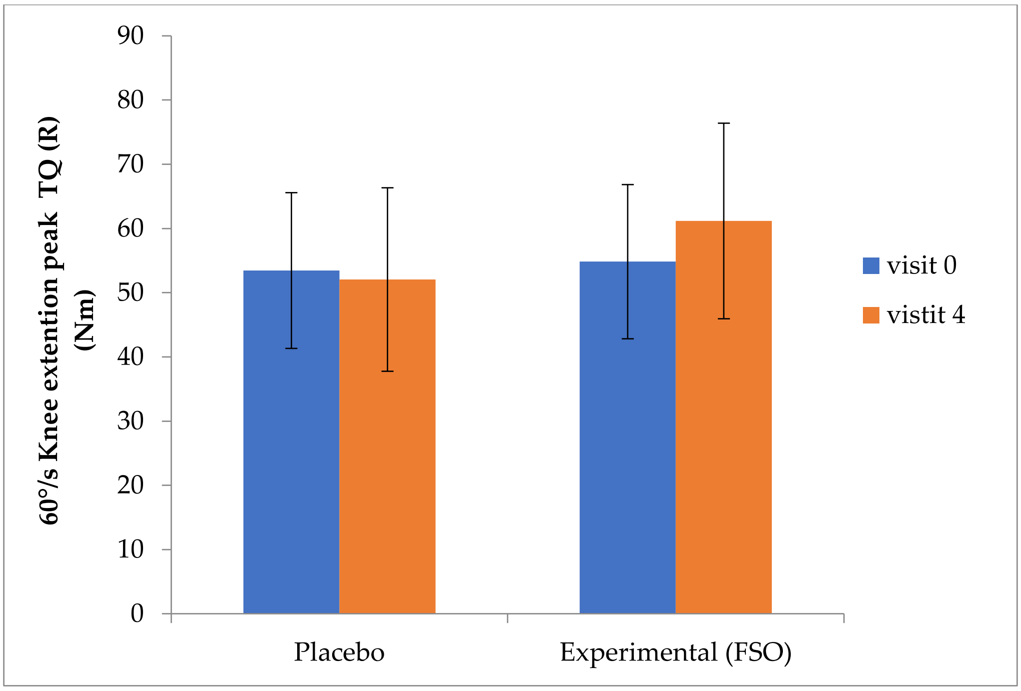

Figure S1. Measurement comparison of the 60º/s right knee extension peak TQ (Nm) in PP population. Each bar represents the mean ± *SD*.

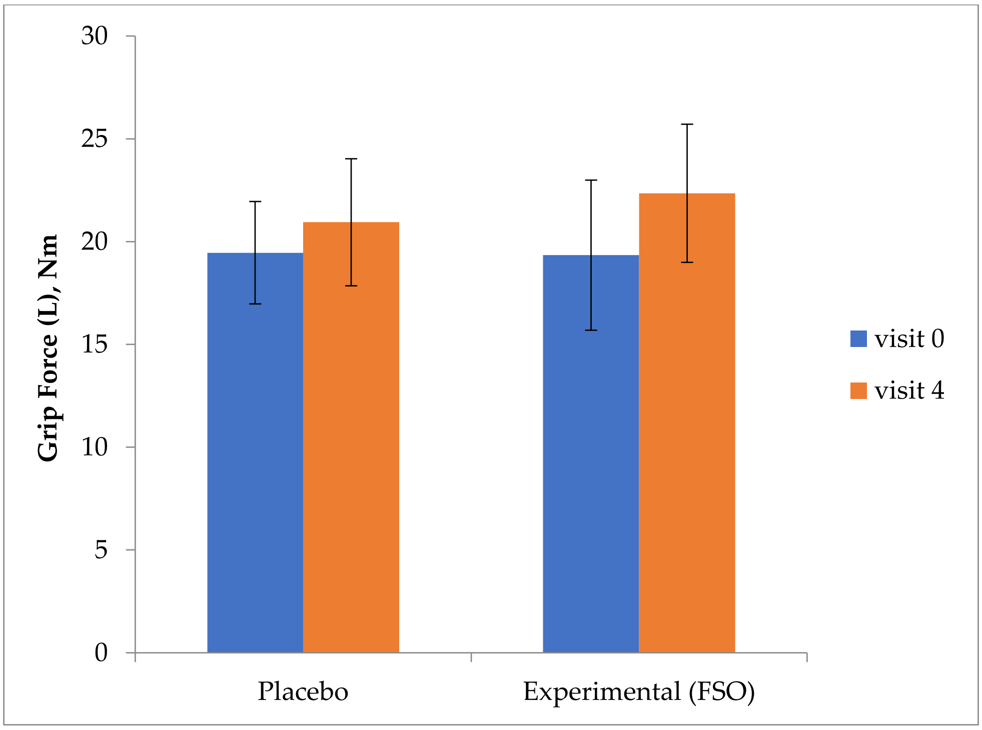

Figure S2. Measurement comparison of the left hand grip force (Nm) in PP population. Each bar represents the mean ± *SD*.

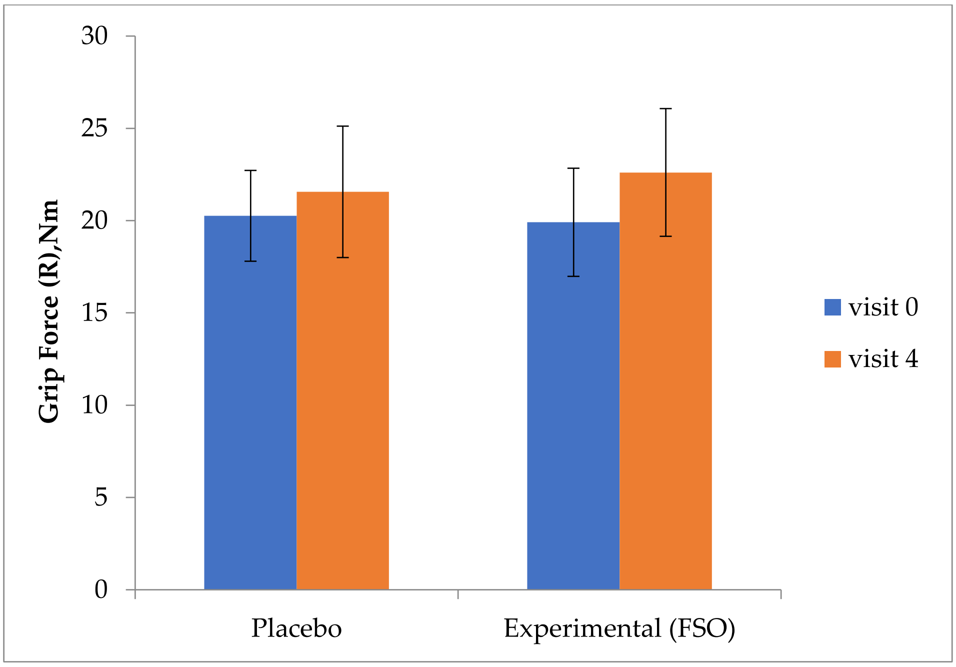

Figure S3. Measurement comparison of the right hand grip force (Nm) in PP population. Each bar represents the mean ± *SD*.

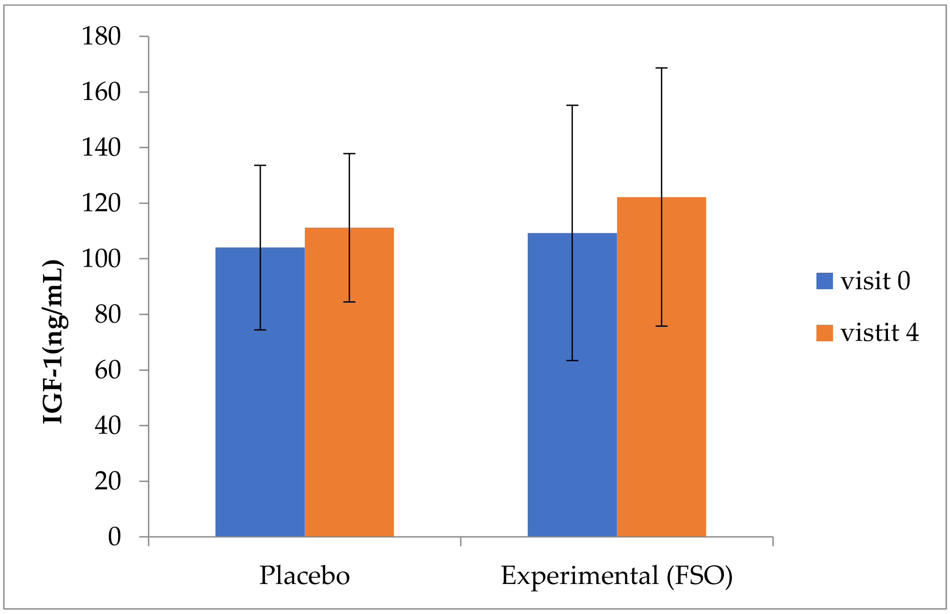

Figure S4. Measurement comparison of IGF-1 level in PP population. Each bar represents the mean ± *SD*.

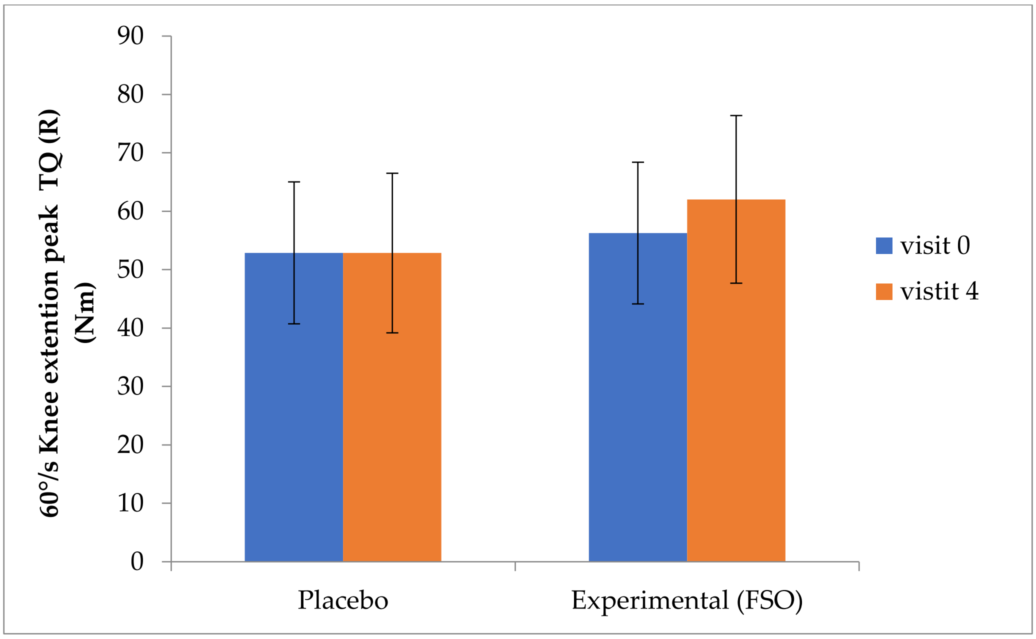

Figure S5. Measurement comparison of the 60º/s right knee extension peak TQ (Nm) in ITT population. Each bar represents the mean ± ***SD.***

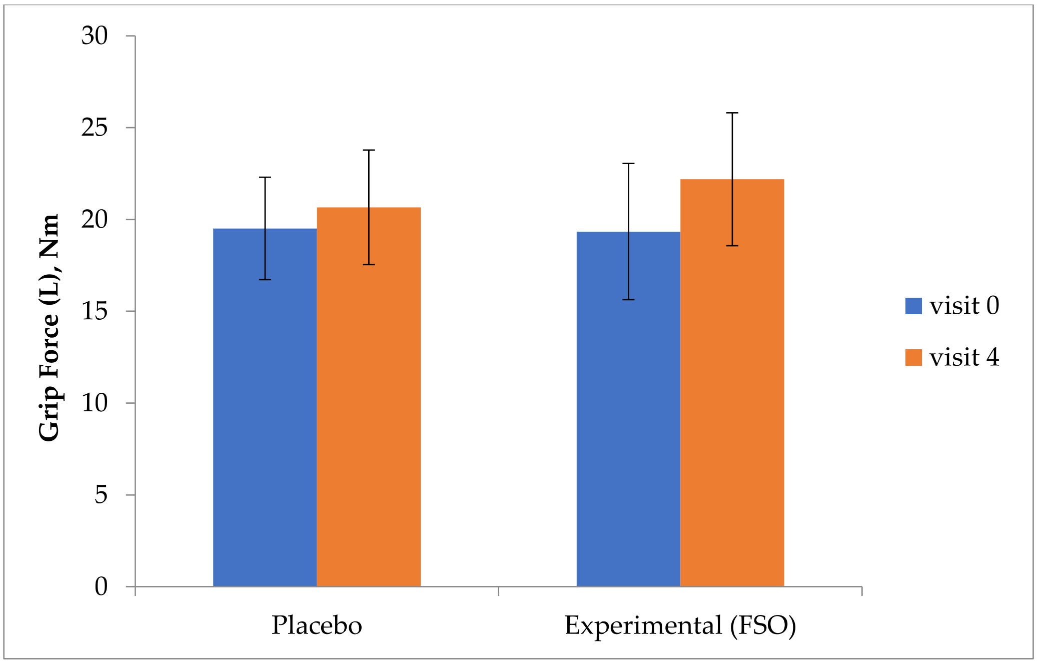

Figure S6. Measurement comparison of the left hand grip force (Nm) in ITT population. Each bar represents the mean ± ***SD.***

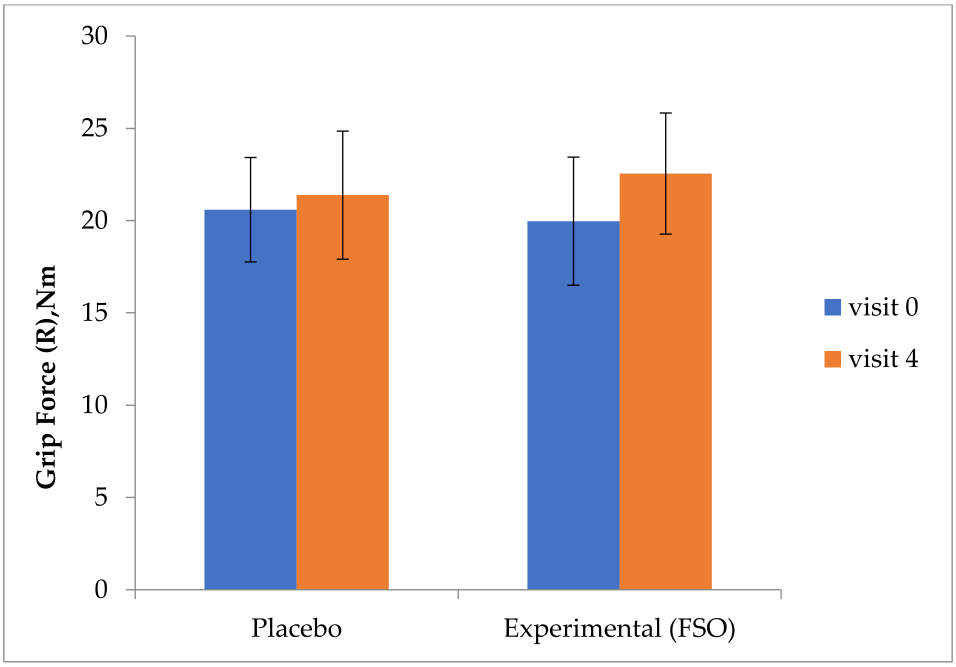

Figure S7. Measurement comparison of the right hand grip force (Nm) in ITT population. Each bar represents the mean ± ***SD*****.**

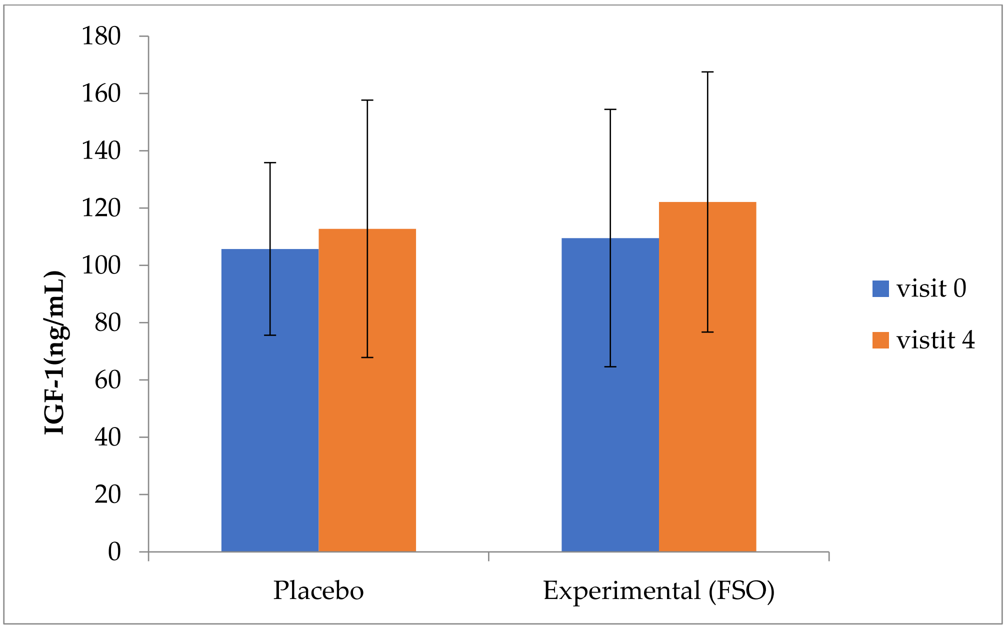

Figure S8. Measurement comparison of IGF-1 level in ITT population. Each bar represents the mean ± ***SD*****.**

Supplementary Table S1. Comparison between and within each group by (PP population)

Variable	Observed value			Change from baseline				
	Control (n=34)	Experimental (n=18)	p value**	Control (n=34)	p value*	Experimental (n=18)	p value*	p value**
**60º/s knee extension peak TQ (R), Nm**								
Visit 2	53.45±12.13	54.83±12.01	0.628	-1.41±10.38	0.736	6.33±11.10	0.15*	0.037*
Visit 4	52.05±14.29	61.17±15.23	0.056					
**60º/s knee flexion peak TQ (R), Nm**								
Visit 2	28.23±9.44	32.50±9.29	0.512	3.63±9.46	0.096	1.50±9.87	0.545	0.477
Visit 4	31.86±11.89	34.00±13.66	0.959					
**180º/s knee extension peak TQ (R), Nm**								
Visit 2	28.64±6.40	32.67±7.67	0.061	2.98±8.83	0.091	1.39±4.43	0.046*	0.530
Visit 4	31.62±9.10	34.06±7.85	0.285					
**180º/s knee flexion peak TQ (R), Nm**								
visit 2	20.95±5.28	25.94±5.97	0.053	1.71±4.69	0.138	-0.78±3.88	0.429	0.094
visit 4	22.67±6.27	25.17±6.10	0.539					
**60º/s knee extension peak TQ (L), Nm**								
Visit 2	57.17±17.03	62.00±14.80	0.310	-2.26±16.42	0.516	-3.74±12.66	0.171	0.734
Visit 4	54.91±12.99	58.26±16.88	0.455					
**60º/s knee flexion peak TQ (L), Nm**								
Visit 2	31.8±12.4	32.3±13.5	0.892	-4.26±7.59	0.013*	-3.74±8.23	0.007***	0.824
Visit 4	27.6±10.7	27.9±12.3	0.919					
**180º/s knee extension peak TQ (L), Nm**								
Visit 2	31.78±9.11	34.22±8.06	0.342	-0.17±6.79	0.903	-1..39±5.79	0.262	0.516
Visit 4	31.61±7.00	32.83±6.57	0.546					
**180º/s knee flexion peak TQ (L), Nm**								
visit 2	22.61±6.32	23.91±5.98	0.476	0.17±5.32	0.640	0.13±3.96	0.876	0.975
visit 4	22.64±5.33	24.04±7.02	0.454					

**ASM/height²**								
visit 2	2.58±0.27	2.65±0.25	0.400	-0.01±0.11	0.695	-0.01±0.13	0.592	0.882
visit 4	2.57±0.26	2.63±0.23	0.415					
**ASM/weight x 100**								
visit 2	10.45±0.75	10.84±1.05	0.152	-0.17±0.46	0.094	-0.11±6.62	0.388	0.729
visit 4	10.28±0.64	10.73±0.92	0.061					
**Total Body Fat**								
visit 2	37.67±4.56	35.40±6.54	0.177	0.70±1.61	0.051	0.87±1.13	0.001***	0.674
visit 4	38.99±4.02	36.56±6.55	0.202					
**Abdominal Fat**								
visit 2	118.4±28.4	106.7±39.8	0.260	9.77±16.39	0.009**	8.93±7.96	0.000***	0.827
visit 4	128.2±25.9	115.7±41.3	0.226					
**Grip Force (L), Nm**								
visit 2	19.46±2.49	19.34±3.65	0.901	1.48±2.31	0.006**	3.00±2.49	0.000***	0.036*
visit 4	20.94±3.09	22.35±3.36	0.146					
**Grip Force (R),Nm**								
visit 2	20.26±2.46	19.91±2.93	0.667	1.60±2.07	0.006**	2.70±1.47	0.000***	0.011*
visit 4	21.56±3.56	22.61±3.46	0.315					
**Pyruvate**								
visit 2	0.545±0.150	0.574±0.163	0.546	0.03±0.20	0.753	0.03±0.17	0.390	0.962
visit 4	0.557±0.122	0.604±0.136	0.229					
**Lactate**								
visit 2	8.17±1.90	9.38±3.70	0.321	1.79±3.38	0.018*	1.34±2.90	0.037*	0.632
visit 4	10.00±3.83	10.72±4.02	0.694					
**IGF-1 (ng/mL)**								
visit 2	104.04±29.58	109.31±45.91	0.646	7.13±25.18	0.188	12.91±23.36	0.015*	0.424
visit 4	111.17±26.67	122.22±46.43	0.329					
**hsCRP**								
visit 2	0.15±0.25	0.16±0.36	0.949	-0.032±0.167	0.371	0.066±0.343	0.369	0.228
visit 4	0.12±0.11	0.22±0.69	0.492					
**EQ-5D-3L**								
visit 2	0.822±0.186	0.798±0.178	0.663	0.049±0.156	0.144	0.061±0.124	0.027*	0.775
visit 4	0.871±0.173	0.859±0.157	0.812					
**VAS**								
visit 2	0.782±0.226	0.771±0.209	0.862	0.061±0.169	0.098	0.074±0.140	0.019*	0.778
visit 4	0.843±0.215	0.845±0.172	0.975					
**IPAQ**								
visit 2	1968.8±1655.9	2216.9±1241.1	0.579	796.36±1746.13	0.053	268.2±1302.9	0.334	0.255
visit 4	2676.6±2393.4	2485.1±1089.4	0.730					
**Dietary Intake**								
visit 2	1699.1±359.8	1562.4±479.8	0.280	-53.19±316.44	0.429	50.72±280.54	0.395	0.245
visit 4	1645.9±410.4	1613.1±512.0	0.812					

The data is expressed as the mean ± SD for pre and post outcomes and comparison between two groups.
* P values were compared within each group.
** P values were compared between groups. Results with statistically significant differences are indicated in bold.

Supplementary Table S2. Comparison between and within each group (ITT population)

Variable	Observed value			Change from baseline				
	Control (n=34)	Experimental (n=18)	p value**	Control (n=34)	p value*	Experimental (n=18)	p value*	p value**
**60º/s knee extension peak TQ (R), Nm**								
Visit 2	52.88±12.15	56.21±12.13	0.320	-0.04±10.13	0.985	5.77±10.58	0.010**	0.048
Visit 4	52.85±13.66	62.04±14.35	0.023*					
**60º/s knee flexion peak TQ (R), Nm**								
Visit 2	28.46±9.24	31.00±9.90	0.344	2.99±9.43	0.118	1.50±9.33	0.607	0.567
Visit 4	31.46±11.86	32.50±13.29	0.768					
**180º/s knee extension peak TQ (R), Nm**								
Visit 2	29.19±6.33	32.98±8.19	0.072	2.64±8.47	0.123	1.88±4.21	0.031*	0.680
Visit 4	31.83±8.61	34.81±8.04	0.206					
**180º/s knee flexion peak TQ (R), Nm**								
visit 2	21.58±5.13	24.92±5.70	0.031*	0.87±5.62	0.429	-1.00±4.18	0.233	0.176
visit 4	22.45±6.31	23.92±6.19	0.403					
**60º/s knee extension peak TQ (L), Nm**								
Visit 2	57.58±16.48	62.27±14.89	0.287	-2.31±15.42	0.368	-4.19±11.98	0.087	0.625
Visit 4	55.27±12.70	58.08±16.84	0.500					
**60º/s knee flexion peak TQ (L), Nm**								
Visit 2	29.73±13.08	31.11±12.83	0.668	-2.88±8.33	0.090	-0.38±5.32	0.230	0203
Visit 4	26.85±10.34	30.31±12.87	0.290					
**180º/s knee extension peak TQ (L), Nm**								
Visit 2	31.27±9.04	34.54±8.35	0.182	0.38±6.66	0.771	-1.42±6.13	0.247	0.313
Visit 4	31.65±7.10	33.12±8.75	0.511					
**180º/s knee flexion peak TQ (L), Nm**								
visit 2	21.92±6.39	23.69±5.76	0.300	0.31±5.30	0.801	0.54±4.34	0.531	0.864

visit 4	22.08±6.02	24.23±6.87	0.241					
**ASM/height²**								
visit 2	2.59±0.26	2.65±0.24	0.428	-0.02±0.11	0.465	-0.01±0.12	0.696	0.839
visit 4	2.58±0.26	2.64±0.22	0.357					
**ASM/weight x 100**								
visit 2	10.40±0.74	10.83±1.03	0.085	0.18±0.46	0.053	-0.10±0.58	0.379	0.580
visit 4	10.22±0.66	10.73±0.91	0.023					
**Total Body Fat**								
visit 2	37.63±4.63	35.05±6.51	0.066	0.66±1.53	0.037*	0.83±1.09	0.001**	0.674
visit 4	38.29±4.65	35.88±6.37	0.074					
**Abdominal Fat**								
visit 2	118.56±34.10	106.85±38.86	0.240	9.77±16.39	0.004***	5.28±27.29	0.000***	0.472
visit 4	128.20±32.11	116.28±40.49	0.240					
**Grip Force (L), Nm**								
visit 2	19.51±2.79	19.34±2.80	0.853	1.15±2.87	0.057	2.85±2.39	0.000***	0.025**
visit 4	20.66±3.12	22.19±3.62	0.112					
**Grip Force (R),Nm**								
visit 2	20.59±2.83	19.97±2.80	0.439	0.79±2.94	0.192	2.58±1.44	0.000***	0.010**
visit 4	21.38±3.47	22.55±3.28	0.221					
**Pyruvate**								
visit 2	0.56±0.16	0.58±0.16	0.785	0.02±0.21	0.651	0.030.651z0.16	0.398	0.852
visit 4	0.55±0.12	0.60±0.13	0.620					
**Lactate**								
visit 2	8.23±1.88	9.62±3.82	0.227	1.58±3.47	0.036*	1.28±2.86	0.039*	0.745
visit 4	9.83±3.83	10.90±4.03	0.486					
**IGF-1 (ng/mL)**								
visit 2	105.75±30.12	109.54±44.92	0.733	7.03±24.64	0.176	11.80±22.77	0.013*	0.422
visit 4	112.77±27.23	122.13±45.47	0.391					
**hsCRP**								
visit 2	0.15±0.25	0.16±0.35	0.830	-0.03±0.16	0.371	0.05±0.33	0.440	0.276
visit 4	0.12±0.11	0.22±0.68	0.463					
**EQ-5D-3L**								
visit 2	0.827±0.187	0.783±0.205	0.426	0.041±0.148	0.169	0.066±0.126	0.014*	0.524

visit4	0.868±0.179	0.849±0.164	0.688					
**VAS**								
visit2	0.790±0.226	0.747±0.254	0.526	0.050±0.162	0.126	0.084±0.155	0.011*	0.454
visit4	0.840±0.220	0.831±0.182	0.868					
**IPAQ**								
visit2	1744.7±1555.0	2155.1±1250.9	0.311	623.8±1551.7	0.061	311.0±1291.4	0.250	0.452
visit4	2451.3±1995.3	2466±1069.5	0.974					
**Dietary Intake**								
visit2	1745.8±373.2	1609.8±477.2	0.258	-47.2±302.0	0.433	61.9±270.1	0.254	0.176
visit4	1698.6±414.4	1671.7±522.6	0.838					

The data is expressed as the mean ± SD for pre and post outcomes and comparison between two groups.
* P values were compared within each group.
** P values were compared between groups.
